# Walnut Consumption May Contribute to Healthy Cardiovascular/Endothelial Function by Maintaining Membrane Integrity

**DOI:** 10.3390/life14111426

**Published:** 2024-11-05

**Authors:** Dora Jarai, Akos Koller

**Affiliations:** 1 Department of Morphology and Physiology, Faculty of Health Sciences, Semmelweis University, 1085 Budapest, Hungary; jarai.dora20@gmail.com; 2 Research Center for Sports Physiology, Hungarian University of Sports Science, 1123 Budapest, Hungary; 3 HUN-REN-SE Cerebrovascular and Neurocognitive Disease Research Group, Faculty of Medicine, Institute of Translational Medicine, Semmelweis University, 1085 Budapest, Hungary; 4 Department of Physiology, New York Medical College, Valhalla, NY 10595, USA

**Keywords:** nutrition, PUFA, phenolic compounds, membrane fluidity, mitochondria, exercise, cardiovascular disease, aging, sedentary lifestyles, mental function

## Abstract

Cardiovascular diseases (CVDs) are the leading causes of death worldwide. A healthy diet has an important role in delaying the development of many modifiable risk factors of CVD, including abdominal obesity, high blood pressure, high plasma levels of cholesterol, and glucose. The consumption of various nuts, especially walnuts, may benefit both primary and secondary prevention due to their bioactive components. This review focuses on (1) the protective role of walnut consumption on CVD at large (2) and the potential cellular and molecular mechanisms by which they have beneficial effects on vascular endothelial function. Walnuts contain many essential ingredients (such as polyunsaturated fatty acids, phenolic compounds, and vitamin E) necessary for the healthy functioning of membranes. Since membranes are involved in nearly all processes associated with life-related function, the main underlying mechanism of walnut-improved cardiovascular function is likely based on improving membrane composition and function by providing all of the substrates necessary for membranes, such as cell, mitochondria, Golgi, nucleus, and so on. In addition to endothelial cell function, all other cells and membranes are likely to benefit from walnut consumption, suggesting that incorporating walnuts into the human diet is essential, for example, during higher physical and mental demand, such as exercise, and may mitigate the risk for the development of cardiovascular diseases and compensate for the sedentary lifestyle, especially in those of an older age.

## 1. Introduction

Cardiovascular diseases (CVDs) are the leading causes of death worldwide [1]. Previous studies have provided ample evidence that a healthy diet has an important role in delaying the development of many modifiable risk factors of CVD, including abdominal obesity, high blood pressure, abnormally high cholesterol and blood glucose levels. According to previous studies (e.g., the Global Burden of Diseases, GBD), nuts and peanuts may be particularly beneficial in both primary and secondary prevention due to their bioactive components, such as unsaturated fatty acids and high amounts of tocopherols [2]. In this review, we are focusing on (1) the protective role of walnut consumption on CVD at large (2) and on the potential cellular and molecular mechanisms by which they have beneficial effects on the vascular endothelial function.

### 1.1. Nuts in Human Diets

Nuts and seeds have been part of the human diet worldwide for thousands of years and were even used in ancient medicinal traditions [1]. Nuts are typically described even as dry fruits with an edible seed and a hard shell [3]. Botanically, they are divided into two main groups: tree nuts and peanuts. Despite peanuts being classified as legumes, they share many nutritional and compositional similarities with tree nuts, such as walnuts (Juglans Regia), almonds (Prunus dulcis), hazelnuts (Corylus avellana), cashews (Anacardium occidentale), and pistachios (Pistacia vera) [1]. Although the consumption of nuts and seeds varies between cultural settings, due to their favorable composition, there is an increasing interest in nut consumption and human health outcomes [4].

### 1.2. Walnuts

As a valuable tree nut, walnut is a well-known member of the Juglandaceae family. The fruit is made up of four individual parts: an outer green husk, the middle shell which must be cracked to release the kernel, a thin skin, and finally, the kernel or meat, which is the most substantial part regarding the nutritional importance of the walnut fruit [5].

## 2. Why Walnuts Among Other Nuts?

Owing to IgE-mediated reactions, nuts may cause allergies in up to 2% of consumers, mostly in children and particularly after the ingestion of peanuts [6]. Significant regional differences in food sensitivities and allergies were observed throughout Europe: while the prevalence of nut and peanut sensitivity was 10–15% in Zurich (CH), it ranged from 5 to 10% in Madrid (E), barely reached 5% in Athens (GR), and in Reykjavik (IS), it did not exceed in 2.5% [7]. Among the three foods that cause allergies the most, hazelnuts were highlighted in three out of five cities, while walnuts were never mentioned [7].

## 3. Demography of Production and Consumption of Walnuts

**Figure 1:**
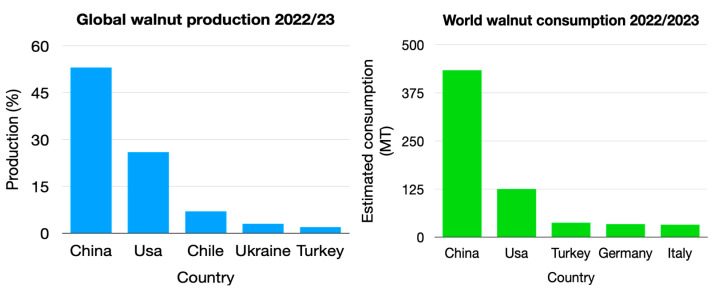
Worldwide production (**left**) and consumption of walnuts (**right**) [8].

These data indicate that the greatest producer and consumer of walnuts is China, followed by the USA, both countries accounting for a significant portion of global production and demand. Global walnut production in 2022/23 reached approximately 1.2 million metric tons (kernel basis), marking the largest volume in the past decade and more than double the crop size from 2012/13. This surge in production reflects increased global demand, likely influenced by the recognized health benefits of walnut consumption (Figure 1).

## 4. Composition Changes in Nuts Due to Processing

Roasting potentially reduces the allergenic effect of certain nuts (such as hazelnuts and almonds) [6]. As a common processing method, roasting also preserves the storability and quality of nuts. Although antioxidants and phenolic compounds may decrease after blanching and peeling (since they reside on the outer peel), roasting has little impact on nutritional composition [3]. Available studies suggest that the effect of roasting on phenolic compounds can vary; however, there is controversial evidence surrounding the temperatures and heating periods [3,6].

## 5. Composition of Walnuts Compared to Other Nuts

Nuts are nutrient-dense foods that contain healthy monounsaturated (MUFA) and polyunsaturated fatty acids (PUFAs), proteins, soluble and insoluble fibers, vitamins E and K, folate, magnesium, potassium, and phenolic compounds [3] (Table 1).

Different fat-soluble bioactive ingredients like fatty acid (FA) content (MUFA and PUFA), magnesium (Mg), tocols (tocopherols and tocotrienols), phytosterols (sterols and standols), sphingolipids, carotenoids, chlorophylls, alkyl phenols, and phenolic compounds (including flavonoids, phenolic acids, tannins, and lignans, among others) all likely contribute to the cardiovascular health-promoting effects of nuts [6] (Table 1).

Table 2 lists the number of phenolic compounds across different types of nuts—walnuts, almonds, cashews, hazelnuts, peanuts, and pistachios—and emphasizes the nutrient density of nuts and their varying phenolic profiles. It shows that cashews and peanuts show comparatively lower phenolic content [6], whereas walnuts contain the highest number of phenolic acids, followed by almonds, hazelnuts, pistachios, and peanuts. Besides phenolic acids, walnuts have the highest hydrolysable tannin content followed by hazelnuts and almonds [6]. According to previous studies, walnuts contain one of the highest levels of antioxidants, such as total polyphenol and tocopherol content among all the studied seeds and nuts, followed by pistachios and hazelnuts [5].

This richness in bioactive phenolics contributes significantly to the cardiovascular health benefits of walnut consumption by improving endothelial function by stimulating NO production, thus enhancing vasomotor regulation, which improves tissue blood flow [6].

This is because proteins in nuts contain large quantities of L-arginine (an important amino acid, 2.28 g/100 g in walnuts), which is a substrate for endothelial nitric oxide synthase (eNOS) responsible for producing vasodilator nitric oxide (NO) [3,9]. Because of this, all tree nuts might have a positive effect on endothelium-dependent vasodilation; thus, their consumption is strongly associated with cardiovascular health [3]. Indeed, the bioactive compounds in tree nuts, including polyphenols are associated with a reduced risk for cardiovascular disease (CVD), type 2 diabetes (T2D), cancer, and all-cause mortality.

Furthermore, research suggests that walnuts may modulate neuroplasticity, provide neuroprotection and promote the vasodilation of brain arteries [10]. Additionally, it has been shown that walnuts have a high concentration of α-tocopherol, a vitamin E family compound that exhibits antioxidant properties principally in the inhibition of lipid oxidation processes [5]. All these findings support our hypothesis that one of the main mechanisms of action of walnuts is to improve the structure and function of membranes of cells, mitochondria and other organelles.

Compared to other nuts, walnuts contain an intermediate amount of fiber; however, available data vary depending on the walnut genotype, the crop year, and the weather conditions [3]. Fibers have a low glycemic index since they are slow-release carbohydrates; as a result, they increase glucose control and potentially decrease cardiometabolic risk [6]. Because of the special mixture of MUFA and PUFA, vitamin E, proteins, soluble and insoluble fibers, and a range of bioactive compounds, such as folate, magnesium, potassium and phenolic compounds, which are essential for the structure of membranes, it is likely that one of the main mechanisms underlying the beneficial effects of walnut consumption is the maintenance and improvement of cell membrane function.

## 6. Membranes Are Life!

Biological membranes are found in all eukaryotic cells and allow life to exist. In addition to separating the interior of the cell from the outside environment, multiple chemical and biochemical actions occur at their surface such as the transfer and conversion of nutrients, synthesis of various molecules, production of energy, and regulation of receptors and channels [11]. Not only the cell but different organelles are surrounded by membranes, for instance, the mitochondria, the Golgi apparatus, the endoplasmic reticulum, and the nucleus as well. The processes necessary to maintain life such as resting membrane potential, action potential, or mitochondrial respiration take place in membranes [12].

### 6.1. Structure and Function of Membranes

The basic structure of cell membranes consists of two layers of lipid molecules generally referred to as a phospholipid bilayer. In addition, various proteins and carbohydrates are key components of membranes [11]. Proteins (such as enzymes, receptors, ion channels, and transporters) are partially or completely embedded into the membrane, helping to maintain structural integrity, aiding the transport of molecules and providing signaling pathways for communications [12]. All these support crucial cellular activities like action potential generation and mitochondrial respiration, making membranes vital for sustaining life.

### 6.2. Phospholipid Bilayer of Membranes and Walnuts/PUFAs

The phospholipid bilayer contains cholesterol as well, which provides flexibility and fluidity. The glycerophospholipids and sphingolipids form the primary structural framework of the membrane, consisting of hydrophilic head groups attached to hydrophobic fatty acid tails, which face inward. Fatty acids in the bilayer vary from saturated (SFA) to monounsaturated (MUFA) and polyunsaturated fatty acid (PUFA) forms, with omega-3 and omega-6 fatty acids playing critical roles in membrane function. Omega-3 fatty acids, such as ALA, found in walnuts, contribute to the hydrophobic core of the membrane. These PUFAs affect membrane rigidity and are essential for processes involving membrane deformation and homeostasis. The hydrophobic tails of these fatty acids point toward each other, forming the inner layer of the membrane. This bilayer structure, along with the lipid composition, profoundly influences biological functions by maintaining membrane integrity and regulating various cellular activities. The lipid profile plays a role in maintaining membrane homeostasis, since PUFAs in glycerophospholipids reduce membrane rigidity and affect processes that accompany membrane deformation [13].

### 6.3. Importance of Omega-3 and Omega-6 Fatty Acid Composition of Membranes

Glycerophospholipids and sphingolipids end in fatty acids, which vary from SFAs to MUFAs and PUFAs, with a large proportion belonging to the inner lipid layer [13]. Both omega-6 (linoleic acid (LA) and arachidonic acid (AA)) and omega-3 (α-linolenic acid (ALA) and docosahexaenoic acid (DHA)) fatty acids are part of the phospholipid bilayer as hydrophobic tails pointing towards each other [13]. Omega-3 and Omega-6 fatty acids are the two classes of essential PUFAs, which means they must be obtained from the diet, and walnut is an excellent source. The difference between the two classes is based on the location of the first double bond of the molecule, counting from the methyl end of the FA. ALA is an n-3 PUFA (the double bond is between the third and fourth carbon atoms) found mostly in nuts and seeds, which is then converted into eicosapentaenoic acid (EPA) and docosahexaenoic acid (DHA), found mostly in fish oils. Conversely, the parent FA of n-6 PUFAs (the double bond is between the sixth and seventh carbon atoms) is linoleic acid (LA; 18:2 n-6), from which a synthesis of arachidonic acid (AA; 20:4 n-6) is made [14]. AA is a well-known precursor of many cellular signaling molecules, such as vasomotor mediators (prostaglandins, thromboxane, 20-HETE, etc.).

### 6.4. The Role of Mitochondrial Membranes

**Figure 2:**
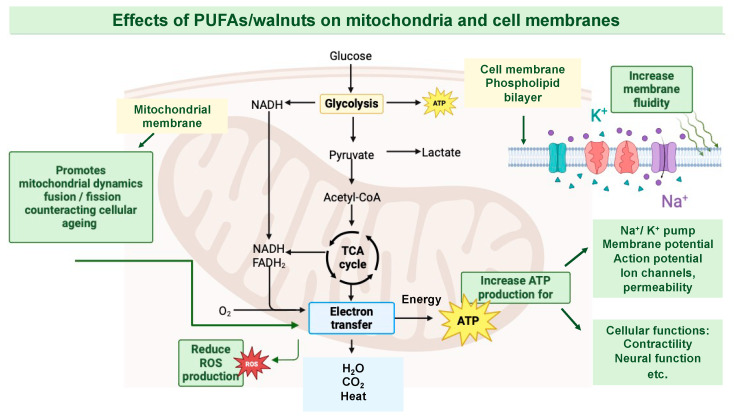
Effects of PUFAs/walnuts on cellular and mitochondrial membrane structure and function contributing to healthy cardiovascular and endothelial function.

The importance of mitochondria has spawned much interest in their role in the aging process and metabolic diseases such as type 2 diabetes. Mitochondria are essential organelles for normal cellular function, particularly regarding their role in energy metabolism. Mitochondrial respiration is a central metabolic process that takes place in biological membranes [15] and involves four main stages: glycolysis, the citric acid cycle, the electron transport chain (ETC), and oxidative phosphorylation [16] (Figure 2).

The hydrolysis of ATP releases free energy to drive processes such as the maintenance of membrane potentials, membrane transport, and protein synthesis. ATP synthesis is driven by a proton gradient across the inner mitochondrial membrane [15].

Dietary lipids in walnuts can likely affect mitochondrial changes associated with aging and disease [15]. The chain enzymes participating in mitochondrial respiration are membrane protein complexes whose activity relies on the composition and fluidity of the mitochondrial membrane lipids [17]. Recent studies have highlighted the role of dietary fats in modulating mitochondrial function with PUFAs, demonstrating their positive effects compared to SFAs [18]. These fatty acids support mitochondrial health by modulating oxidative stress, improving membrane fluidity and possibly reducing excessive mitochondrial fission [18]. Additionally, n-3 fatty acids and conjugated linoleic acid (CLA) have gained special attention for their potential role in supporting mitochondrial adaptation and function, particularly by improving energy metabolism and enhancing mitochondrial resilience [18]. These findings suggest that walnuts may therefore contribute to mitochondrial health not only by reducing oxidative stress but also by enhancing mitochondrial adaptations like those induced by physical exercise, further supporting cardiovascular and metabolic health.

### 6.5. Walnut Promotes Membrane Fluidity

While lipids with long saturated fatty acids, like sphingolipids, make membranes less fluid due to their stronger lipid–lipid interactions, unsaturated fatty acids do the opposite and increase membrane fluidity [13]. Walnut PUFAs in glycerophospholipids (such as DHA) may decrease membrane bending rigidity consequently, helping cellular processes, like endocytosis, where membrane deformation occurs [13]. One proposed mechanism through which n-3 PUFAs provide protective effects is their capacity to significantly alter cell membrane properties when integrated into the phospholipid bilayer and regulate membrane ion channels to prevent arrhythmias, including malignant ones [19].

These characteristics suggest that unsaturation in acyl chains increases membrane fluidity; thus, the level of unsaturation of lipids in the membrane might affect its organization [13].

Walnuts contain—more than any other nuts—many essential ingredients (such as PUFAs, phenolic compounds, and vitamin E) necessary for the healthy functioning of membranes. Healthy membranes are crucial for various cellular functions including nutrient transport, signaling and energy production, all of which are essential for both physically active and inactive lifestyles [13]. Walnut consumption supports membrane integrity, helping to maintain cellular function, which is particularly important in reducing the risk of chronic diseases, such as cardiovascular issues, by supporting vascular health and improving lipid profiles. Incorporating walnuts into the diet regardless of activity level plays a vital role in enhancing overall health by promoting healthy membranes, which are essential for sustaining life [14].

## 7. The Role of Walnut Consumption: Membrane Lipids in Health and Disease

Many diseases are related to lipid composition changes, like mutations in lipid synthesis or small differences in lipid chemical structures because of decreasing membrane fluidity [13]. For instance, in a study, rats with type 2 diabetes were supplemented for 5 months with ALA and physiochemical properties of mitochondrial membranes, and the consequences of mitochondrial respiration were observed. While diabetes increased the proportion of SFAs, decreased membrane fluidity (they used fluorescent probe dyprenylpropane), and decreased mitochondrial respiration, ALA supplementation partially kept the physicochemical properties regulated and reduced blood glucose levels gradually by increasing insulin sensitivity [20]. Walnuts contain high amounts of omega-6 and omega-3 polyunsaturated fatty acids compared with other nuts (Table 3). Several mechanisms suggest that these fatty acids have an antiarrhythmic effect by modulating ion channels, such as an antilipemic effect by lowering blood lipid levels and an antithrombotic effect by inhibiting platelet aggregation and lowering the synthesis of thromboxane [21]. Furthermore, studies show that the frequent consumption of walnuts favorably modified the lipoprotein profile and decreased serum cholesterol [22].

Table 3 shows the FA content (g/100 g) for various nuts including walnuts, almonds, cashews, hazelnuts, peanuts, and pistachios. Walnuts contain significantly higher levels of PUFAs (49.3 g/100 g) compared to the other nuts, making them a standout source of these essential fatty acids. In contrast, peanuts, pistachios and almonds have moderate PUFA content at 15.6 g/100 g, 14.4 g/100 g and 11.2 g/100 g, respectively. Hazelnuts and cashews are on the lower end of the spectrum, with PUFA levels of 7.9 g/100 g and 7.8 g/100 g [23].

### 7.1. Cellular Mechanisms of Polyunsaturated Fatty Acid Content of Walnuts on Endothelial Function

From the standpoint of CVD prevention, n-3 PUFAs are the more important class of essential FAs. Studies focusing on the cardiovascular effects of n-3 PUFAs found that ALA might reduce the risk of clinical events of CVD with an atherosclerotic origin, whereas its metabolites, EPA and DHA have a negligible effect [19].

Walnuts are a uniquely rich source of ALA and epidemiological studies suggest that plant-derived ALA may confer cardiovascular benefits [24] by reducing low-density lipoprotein cholesterol (LDL-C) and triglycerides levels in the plasma, thereby contributing to the improvements of the vasomotor (typically measured through NO production and endothelial-dependent vasodilation) function of the endothelium [19]. Their role in the prevention of CVD may start with the changes in the cell membrane because of n-3 PUFA incorporation [14], lowering membrane cholesterol. The poor incorporation of cholesterol into n-3 PUFA (EPA and DHA)-containing phospholipid bilayers suggests the incompatibility of a highly unsaturated lipid environment and cholesterol. In addition, ALA may also modulate cholesterol partitioning into membranes [25].

A study involved 20 hypercholesterolemic individuals (age 49.3 ± 1.7) and demonstrated that a diet rich in alpha-linolenic acid (ALA) from walnuts, walnut oil, and flax seeds significantly improved flow-mediated dilation (FMD) by 34%, reduced diastolic blood pressure by 2–3 mm Hg, and lowered total peripheral resistance by 4%. These effects were seen both at rest and during acute stress, underscoring the cardiovascular benefits of ALA from walnuts in hypercholesterolemic individuals [26]. In another study, 18 healthy men participated in a diet program where 20 percent of the calories of the diet were derived from walnuts. It was found that incorporating moderate quantities of walnuts into the recommended cholesterol-lowering diet while maintaining the intake of total dietary fat and calories decreases serum levels of total cholesterol and favorably modifies the lipoprotein profile [27].

In a study where streptozotocin-induced diabetic rats were treated with vehicle (0.01% alcohol) or ALA (500 µg/kg per day) for 4 weeks, there was a significant improvement in concentration-dependent relaxation on aortic segments to the endothelium-dependent, NO-mediated responses to acetylcholine (ACh). Also, ALA inhibited endothelial inflammation as indicated by the decreased level of soluble P-selectin and intracellular adhesion molecule-1 (ICAM-1) in the vascular wall of diabetic rats. These data indicated that ALA has cardiovascular protective effects, but the underlying mechanisms remain largely unknown [28]. Another study involved 27 overweight individuals who followed either a walnut or almond diet. The walnut diet significantly improved flow-mediated dilation (FMD) (*p* = 0.004) and reduced soluble vascular cell adhesion molecule (sVCAM) levels (*p* = 0.009), which are key markers of endothelial function and vascular inflammation [29].

Information about molecular targets of PUFAs focuses on Ca channels mediating vasodilation such as transient receptor potential vanilloid 4 (TRPV4) channels. These channels play a role in endothelial and smooth muscle cell activation and decreasing systemic blood pressure. By increasing endothelial intracellular Ca^2+^, releasing nitric oxide (NO) and subsequently causing smooth muscle cell hyperpolarization, n-3 PUFA-like molecules may have an antihypertensive effect through TRPV4 channels, although it is not clear whether all n-3 FAs have these features [19]. In vivo testing in a transgenic worm expressing rat TRPV4 showed that EPA and its eicosanoid derivative are essential for the function of this channel.

### 7.2. Role of Consumption of Walnuts in the Prevention of Cardiovascular Disease

Due to their fat-soluble bioactive compounds such as fatty acid (FA) content and phenolic compounds (Table 2 and Table 3), walnuts are ideal dietary supplements for improving vascular health. Previous studies have shown that a higher consumption of walnuts reduces mediators of oxidative stress [4] (such as superoxide and hydrogen peroxide peroxy-nitrite), inflammation, hyperglycemia, insulin resistance, and endothelial dysfunction, thereby reducing the likelihood of metabolic syndrome (MetS) through modest decreases in triglycerides and fasting blood glucose [4]. A study involving 46 overweight adults (average age 57.4 years; 28 women and 18 men) with metabolic syndrome found that the daily consumption of 56 g of walnuts significantly improved flow-mediated dilation (FMD), a marker of endothelial function, without causing weight gain. Beneficial trends in systolic blood pressure were also observed. These findings suggest that walnuts can have a positive impact on cardiovascular health, especially in populations at higher risk for diabetes and cardiovascular diseases [24].

Moreover, a randomized controlled crossover trial involving 45 men and women (aged 30–65) at risk for cardiovascular disease showed that a walnut-enriched diet (incorporating 57–99 g/d (i.e., 18% of energy) of walnuts as a snack into a healthy dietary pattern) significantly lowered central diastolic blood pressure compared to an oleic acid-replaced ALA diet. The walnut diet also reduced mean arterial pressure and improved lipid profiles, highlighting the unique benefits of consuming whole walnuts beyond their fatty acid content [30].

A study that aimed to assess the immediate effect of a polyphenol-rich meal (75% energy from walnuts or almonds) on plasma polyphenol, antioxidant capacity, and lipid peroxidation in healthy participants found that plasma polyphenol was significantly increased following both nut meals, the walnut meal having a more sustained higher concentration than the almond meal [31].

In addition to their low-density lipoprotein cholesterol (LDL-C) lowering effect, a cause-and-effect relationship was also found between walnut consumption and endothelium-dependent vasodilation (for which ultrasound measurements of brachial artery vasomotor function were taken), which characterizes healthy vascular function [32].

In a study written by Ros et al., 21 adults (with high cholesterol levels) were randomized to a cholesterol-lowering Mediterranean diet and a diet of similar energy and fat content, in which walnuts replaced 32% of the energy from monounsaturated fat. Participants followed each diet for 4 weeks. As a result, endothelial function assessed by the flow-mediated dilation of the brachial artery with ultrasound showed a greater improvement in the walnut group and significantly reduced total cholesterol and LDL cholesterol and reduced levels of vascular cell adhesion molecule-1 [9]. Because nuts are also rich in phytosterols and their molecular structures are like that of cholesterol, phytosterols inhibit the intestinal absorption of cholesterol, consequently lowering plasma cholesterol and LDL levels [22].

Epidemiological studies indicate that there is a dose–effect relationship between the daily intake of nuts and CVD risk reduction, with walnuts showing the strongest association [33]. In 11 cohort comparisons (including 376,228 participants), the high consumption of nuts was associated with a lower risk of CVD mortality; however, it was also found that the optimal intake might be 15–20 g/day, and above that (up to 28 g/day), the benefits were limited [32]. Available evidence is controversial regarding the optimal amount of intake since a study where the absorption of gamma-tocopherol was measured showed that the consumption of 75 g/day of walnuts improved vascular status (a decrease in the α-T: γ-T ratio with an increase in serum γ-T) and biomarkers of prostate (PSA) in men at risk for prostate cancer [4].

## 8. Potential Role of Walnuts/PUFAs in Athletes and Elderly

### 8.1. Post-Exercise Recovery of Athletes

Improved endothelial function potentially allows athletes to maintain better cardiovascular function and sustain higher levels of athletic performance [34,35]. By increasing NO production, omega-3 fatty acids, particularly EPA and DHA (converted from ALA), promote vasodilation and enhance blood flow, therefore increasing oxygen uptake and delivery to muscles and supporting aerobic metabolism [34]. The presence of antioxidants alongside unsaturated fatty acids found in walnuts might help attenuate oxidative stress in athletes, thus improving muscular performance and immune function [35]. The anti-inflammatory properties of omega-3s help reduce exercise-induced muscle soreness and damage (by reducing the production of pro-inflammatory cytokines and ROS as well as increasing anti-inflammatory mediators), allowing athletes to recover more efficiently after intense workouts [34]. Indeed, previous studies showed that high PUFA content in walnuts, particularly omega-3 fatty acids, supports membrane fluidity, reduces inflammation, and enhances recovery after exercise [36]. Thus, by incorporating walnuts into the diet, both athletes and amateurs can utilize these benefits to support endurance, reduce inflammation, and enhance recovery, but these potential benefits depend on several factors, including the intensity of physical activity and dietary habits [37]. Nevertheless, further studies should aim to elucidate specific benefits and the underlying mechanisms.

### 8.2. Effects of Walnut/PUFAs Supplementation in Elderly People

A two-year randomized dietary intervention trial involving free-living elderly adults (aged 63–79) demonstrated that incorporating walnuts into their daily diet (15% of energy intake) significantly increased the intake of essential nutrients, such as vegetable protein and polyunsaturated fatty acids (PUFAs) while lowering saturated fat and sodium intake [38]. These findings suggest that walnut consumption can contribute to healthy aging by correcting nutritional deficiencies often seen in elderly. This study provides further evidence of the role of walnuts in improving cardiovascular and overall health, particularly in aging populations, which may compensate for the sedentary lifestyle often present in older-aged individuals.

The efficacy of n-3 PUFA therapy was studied in a randomized controlled trial, showing that it slowed age-related declines in muscle mass and function, which are well-known risk factors for impaired physical performance and independence in older adults. In this study, sixty healthy men and women aged 60–85 years received n–3 PUFA (n = 40) or corn oil (n = 20) for 6 months. After 6 months, the n–3 PUFA group showed significant increases in thigh muscle volume (3.6%), handgrip strength (2.3 kg), and one-repetition maximum muscle strength (4.0%) compared to the control group. There was also a trend toward increased isokinetic power (5.6%; *p* = 0.075) [39].

Moderate walnut intake affects lipid profiles, steroid hormones, and inflammation in physically active elderly men [40]. The randomized controlled trial involved 20 healthy elderly males divided into two groups: one consumed 15 g of walnuts daily alongside regular training, while the other group adhered to a control diet with identical training. The findings indicated significant improvements in the walnut-consuming group, including elevated HDL levels and reduced total cholesterol, LDL, and triglycerides. Furthermore, the walnut group demonstrated greater increases in testosterone levels and decreases in cortisol and C-reactive proteins compared to the control group, suggesting that walnut supplementation could enhance lipid profiles and hormonal equilibrium and mitigate inflammation [40].

In a study written by Kevin et al., involving older adults, 16 weeks of mixed nut consumption (60 g/day mixed nuts: walnut, pistachio, cashew and hazelnut) significantly improved endothelial function, as measured by brachial flow-mediated dilation (*p* < 0.001), along with reductions in arterial stiffness and improvements in cerebral blood flow [41]. These findings may suggest that walnut consumption by sedentary and/or elderly people may also compensate for the adverse effects of an inactive lifestyle.

The walnut-improved membrane function may contribute to the maintenance and improvements of brain function [42,43]. For example, walnut consumption dose-dependently improved the cognitive function in aged rats [42] and the memory deficits and learning skills in a transgenic mouse model of Alzheimer’s disease [43]. These benefits are attributed to walnuts’ high content of polyunsaturated fatty acids (PUFAs) and phytomelatonin, which regulate cerebral processes and sleep (with an average content of 350 ng/100) [44].

In a study using a mouse model of Alzheimer’s disease (AD), long-term walnut supplementation significantly enhanced memory, learning, and motor coordination and reduced anxiety. It also mitigated amyloid-beta (Aβ)-induced oxidative stress and related cell death [45]. These findings suggest that early and sustained walnut consumption may help preserve cognitive function and protect against cognitive decline and AD.

The WAHA (Walnuts And Healthy Aging) study, a two-year, observer-blinded, randomized controlled trial conducted at Loma Linda University, USA, and Hospital Clínic, Barcelona, Spain, investigated the effects of a diet enriched with walnuts (providing 15% of daily energy intake) compared to a walnut-free diet on cognitive function in healthy elders aged 63–79. Although the walnut supplementation over two years did not prevent cognitive decline in the overall group of cognitively healthy elderly participants, subsequent analyses indicated that participants from the Barcelona site exhibited enhanced global cognition and perception scores compared to the control group. This benefit was corroborated by brain fMRI findings in a subset of participants [46].

## 9. Conclusions

Walnuts contain—more than any other nuts—many essential ingredients (such as polyunsaturated fatty acids, phenolic compounds, and vitamin E) necessary for the healthy functioning of membranes. Since membranes are involved in nearly all processes associated with life-related function, the main underlying mechanism of walnut-improved cardiovascular function is likely based on improving membrane composition and function by providing all of the substrates necessary for membranes, such as cell, mitochondria, Golgi, nucleus, and so on. In this review, we focused on endothelial cell function, but all other cells and membranes likely benefit from walnut consumption.

Thus, incorporating walnuts into the human diet is essential to mitigate the risk of developing cardiovascular diseases. Because of this, walnuts should be considered a valuable additive to a regular diet, especially in those of an older age and during higher physical and mental demands, such as exercise. Nevertheless, further research is still needed to elucidate further the specific molecular pathways responsible for the beneficial effects of walnuts and the optimal dietary recommendations for maximizing the health benefits of walnut consumption.

## Figures and Tables

**Table 1:** The main composition of various nuts [3].

Nut	Lipid (%)	Lipid (PUFA g/100 g)	Protein	Vitamin E	Minerals—Mg (mg/100 g)	Fiber (%)
Walnut	64.5–65.2	47.174	14.4–16.0	0.1–13.0	158–201	6.7
Almond	43.3–50.6	12.329	16.8–25.4	2.4–25.9	275	11.8–13.0
Cashew	42.8–43.9	7.845	17.5–19.0	0.0–0.9	292	1.4–3.3
Hazelnut	59.8–61.5	7.920	14.5–15.2	3.5–15.0	140–163	3.4–9.7
Peanut	49	33.0	25.8	0.4	168–173	9.0
Pistachio	44.4–45.4	14.380	19.4–22.1	0.3–2.3	117–121	10.3

**Table 2:** Number of phenolic compounds in various nuts [6].

Nut	Phenolic Acid	Tannins	Flavonols	Anthocyanins	Stilbenes
Walnut	**20**	**19**	**6**	**1**	**0**
Almond	15	2	11	4	2
Cashew	3	0	0	0	0
Hazelnut	14	3	3	0	1
Peanut	10	0	2	0	1
Pistachio	13	0	7	5	1

**Table 3:** SFA, MUFA and PUFA content of various nuts (source: [23]).

Nut	SFA	MUFA	PUFA
Walnut	**6.1**	**9.6**	**49.3**
Almond	3.8	31.3	11.2
Cashew	7.8	23.8	7.8
Hazelnut	4.5	45.7	7.9
Peanut	6.3	24.4	15.6
Pistachio	5.9	23.3	14.4

## Data Availability

Not applicable.
